# Prevalence of Anger in Medical Students: A Tertiary Care Experience from a Developing Country

**DOI:** 10.7759/cureus.4258

**Published:** 2019-03-16

**Authors:** Syed Ijlal Ahmed, Saher Naseeb Uneeb, Syeda Beenish Bareeqa, Sharmeen Ibrahym, Sindhu Muneer, Syed Hasham Humayun, Syeda Sana Samar

**Affiliations:** 1 Internal Medicine, Liaquat National Hospital and Medical College, Karachi, PAK; 2 Medical Education and Simulation, Liaquat National Hospital and Medical College, Karachi, PAK; 3 Oncology, Jinnah Medical and Dental College, Karachi, PAK; 4 Medical Education and Simulation, Liaquat National Hospital and Medical College, Karachi , PAK; 5 Miscellaneous, Jinnah Medical and Dental College, Karachi, PAK; 6 Internal Medicine, Jinnah Sindh Medical University, Karachi, PAK

**Keywords:** medical student, behavior, anger, prevalence

## Abstract

Background: Anger is defined as an emotional state that involves displeasure and consists of subjective feelings that vary in intensity, from mild irritation or annoyance to intense fury and rage. Anger is dangerous because it affects the health of the individual. It also affects relationships between fellow physicians and nurses and can ultimately compromise patient care. Medical school is perceived as stressful and a number of studies have proved the high prevalence of anxiety, stress, and depression in medical students. However, no significant studies have been performed to assess the frequency of anger in medical students. The purpose of our study is to find out whether anger is prevalent among medical students and its effect on different aspects of student’s lives.

Methods: A cross-sectional survey was conducted on the students from all five years of Liaquat National Medical College. Sampling technique used was nonprobability purposive sampling. A self-administered questionnaire was filled by medical students. Data were recorded and analyzed using the IBM statistics SPSS software.

Results: A total of 205 students participated in the survey. Using the questionnaire, it was found that the highest frequency of anger was found in first-year students (97.5%), followed by the fourth year (97.4%), final year (97.2%), third year (95.7%), and second year (91.9%). All five years identified stress as a major predictor of anger. Anger had the greatest effect on decision making, especially in final year medical students.

Conclusion: Authors concluded a high frequency of anger in medical students. Increased stress has negative impacts on the mental health and coping strategies of students which greatly affect their decision-making power.

## Introduction

Anger refers to feelings and represents the emotional or affective component of at least of some kinds of aggressive behavior [1]. Anger is hazardous to the individual because it affects health and is a risk factor for cardiovascular diseases [2-3]. Anger also affects relationships between fellow physicians and nurses [4]. This can ultimately compromise patient care [5].

Medical education is perceived as being stressful. A number of studies have been conducted on the prevalence of anxiety, stress, and depression among medical students [6-7]. These studies have reported that there is significant distress among medical students. One of the causes of stress has been identified is higher medical student year [8]. Another cause is not being able to indulge in recreational and social activities [9]. Increase in workload and the pressure of learning professional knowledge and skills was another factor that was cited to contribute to stress [10]. Exams and continuous assessments have also been considered as an important factor that contributes to psychological distress in medical students [6].

Similar to anger, stress and anxiety have detrimental effects on the health of the individual by acting as a risk factor for cardiovascular disease [11]. Not only that but they also lower patient care [12].

Previous studies in Pakistan have also shown a higher prevalence of anxiety and depression in medical students [13]. Causes of stress include examinations and a greater amount of material that needs to be memorized [14]. Other causes include the inability to cope, psychological pressure, and mental tension [15].

Even though studies have been done on the prevalence of stress, anxiety, and depression among medical students, no studies have been done on the prevalence of anger. The purpose of our study is to find out if anger is prevalent among medical students because anger just like all the other psychological stressors is not only detrimental for the individual involved but also for the patients that medical students will have to deal with in the future.

## Materials and methods

Our study was a cross-sectional survey conducted among the students of the tertiary care center of a developing country. The sample size was calculated and the sampling technique used was nonprobability purposive sampling. A self-administered questionnaire was filled by 197 medical students. The students were asked to fill the questionnaire containing 13 questions. The designed questionnaire comprises the following questions:

-Do you feel angry? (never/sometimes/most of times/always)

-What makes you angry? (stress/burden of studies/failure/quarrels/none)

-Do you feel hostile towards others? (never/sometimes/most of the time/always)

-Do you think your anger is affecting your relations? (never/sometimes/most of the time/always/not sure)

-How is your behavior with your colleagues? (Good/average/bad)

-How much time do you spend studying daily? (don’t study daily/less than one hour/one to two hours/two to four hours/four to six hours)

-How much time do you daily spend with friends and family? (less than one hour/ one to two hours/ two to four hours/four to six hours/six to eight hours/ eight to ten hours)

-Do you get irritated? (Never/sometimes/most of times/always)

-Do you shout at people? (Never/sometimes/most of times/always)

-Does your anger affect your ability to make decisions? (never/sometimes/most of times/always)

-Does your anger affect your sleep? (never/sometimes/most of times/always)

-Does your anger affect appetite? (never/sometimes/most of time/always)

-Does your anger interfere with your physical health? (never/sometimes/most of time/always)

These questions were formulated after a thorough literature search and ‘Becks depression inventory’ was used as a reference questionnaire. The inclusion criterion was the inclusion of all healthy undergraduate students of the medical college. The exclusion criterion was the exclusion of graduate students. The data were recorded and analyzed using the IBM SPSS vs21. The variables were analyzed for descriptive and inferential statistics including the chi-square test.

## Results

A total of 205 students participated in the study. The data showed that medical students of all five years displayed a high frequency of anger (>90%). Majority of people from all five years identified stress as a major cause of their anger. Along with anger there was also increased irritability (>84 %) and increased efficiency on decision making (>68 %). Anger also affected relationships to a certain degree and caused an increased tendency for people to shout (>55%). Anger had little effect on sleep (<62.2%), appetite (<70.3%), and physical health (<65.3%). Even though the anger frequency was quite high (>90%), the hostility frequency was low (<76 %). Among the medical students the third years despite showing a slightly lower anger frequency (95.7%) compared to other years, showed significantly higher hostility (73.9%) and higher tendency to shout at people (80.4%). These patterns had a greater effect on their relationships (78.3%) and their physical health (65.2%). Table 1 shows the frequency of anger and its effects on daily life.

**Table 1:** Anger frequency and its effects on different aspects of daily life.

Medical year	Anger frequency (%)	Hostility (%)	Effect on relationships (%)	Easily irritated (%)	Shout at people (%)	Affects decision making (%)	Affects sleep (%)	Affects appetite (%)	Affects physical health (%)
1st Year (n = 40)	97.5	75	65	92.5	55	70	40	52.5	42.5
2^nd^ Year (n = 37)	91.9	67.6	70.3	97.3	59.5	72.9	62.1	70.2	62.1
3^rd^ Year (n = 46)	95.7	73.9	78.3	95.6	80.4	71.7	50	65.3	65.2
4^th^ Year (n = 38)	97.4	52.6	55.3	84.3	68.4	68.5	42.1	47.4	39.5
4^th^Year (n = 36)	97.2	58.4	74.9	97.2	72.2	83.4	50	44.4	52.8

Regarding the study schedule of students, all five years displayed a high anger frequency; however, majority said they did not study daily (except for third years who studied two to four hours daily) and spent two to four hours daily with family and friends. Majority of the medical student years did not study daily. Third years who showed the highest hostility spent the most amount of time studying (one to two hours). First-year students who had the greatest anger frequency studied longer than other medical years (four to six hours). Table 2 shows the study schedule of medical students.

**Table 2:** Study schedule of medical students.

Medical Year	Don’t study daily (%)	Less than one hour (%)	1-2 hours (%)	2-4 hours (%)	4-6 hours (%)
1st Year (n = 40)	27.5	7.5	25.0	27.5	12.5
2^nd^ Year (n = 37)	37.8	16.2	16.2	18.9	10.8
3^rd^ Year (n = 46)	21.7	21.7	30.4	23.9	2.2
4^th^ Year (n = 38)	50.0	15.8	13.2	18.4	2.6
5^th^ Year (n = 36)	50.0	8.3	27.8	8.3	5.6

## Discussion

Medical education is perceived as stressful. A number of studies have been conducted on the prevalence of anxiety, stress, and depression among medical students. These studies have shown that there is a high prevalence in medical students and causes significant psychological distress [6]. Our study suggests that there is a high prevalence of anger in medical students of all five years.

One of the major causes of anger was cited as stress. This is in concurrence with a number of studies that suggest that medical students face a significant amount of stress in medical school [7, 9].

The greater amount of stress was related to higher medical student year [8]. Our study, however, showed that the anger frequency was significantly high in all five years but it was comparatively lower in second-year medical students. In Pakistan, a study was conducted on ‘Prevalence of anxiety and depression among medical students’ at Nishtar Medical College [15]. This study suggested that second-year medical students were more likely to be depressed and anxious followed by third-year medical students. Another study conducted in Pakistan ‘Prevalence of anxiety and depression among medical students’ [14] also revealed that anxiety and depression were significantly higher in first- and second-year students. Our study, however, suggests that second-year students had the least anger frequency while first-year students had the most anger frequency.

A study conducted on ‘Prevalence and socio-demographic co-relations of depression, anxiety and stress' [16] suggested that students who were satisfied with their education had lower depression, anxiety, and stress. Our study showed that the majority of students in all five years did not study daily. This could be a contributing factor that leads to decreased academic satisfaction and can increase stress levels which can increase anger frequency.

A study conducted on ‘Perceived stress during undergraduate medical training’ [10] suggested that an increase in workload is one of the major causes of stress. In our study, the third-year medical students who displayed the greatest amount of hostility had the majority of students who studied daily (one to two hours). This could be because of the increase in workload that leads to stress.

First-year students who had the greatest anger frequency studied longer than other medical years (four to six hours). This could lead to an increase in stress which could be a reason for the higher anger frequency.

There was another study which showed ‘Prevalence of anxiety, depression and other factors associated with medical students in Karachi’ [13]. This study suggested that there are certain risk factors other than academics that predispose to anxiety and depression. Even though our study did not look into another factor for psychological distress it was found that academics can be a possible cause of distress.

Although the anger frequency was high, it had little impact on appetite, sleep, and physical health. Third years displayed the greatest hostility and that could be the reason why they had the highest effect of anger on physical health.

A study “The terrible twos ‘anxiety and anger’” [2] has suggested that anxiety and anger increase lipid levels, cardiovascular diseases, and pain. Anger and stress also can compromise patient care [5, 12]. Another study was done on ‘Psychological stress and treatment’ [17] and how stress and anger should be treated with psychotherapy.

These studies suggest that anger and anxiety are not only hazardous to health but they also affect patient care. Specific measures should be taken to reduce them.

## Conclusions

We conclude that there was a high frequency of outrage and anger among medical students and it affects their life in different aspects. Further studies should be done on this issue to devise interventions which help to reduce stress and its consequences on medical students.
